# Endoscopic vs. microscopic transsphenoidal pituitary surgery: a single centre study

**DOI:** 10.1038/s41598-020-78823-z

**Published:** 2020-12-14

**Authors:** Morten Winkler Møller, Marianne Skovsager Andersen, Dorte Glintborg, Christian Bonde Pedersen, Bo Halle, Bjarne Winther Kristensen, Frantz Rom Poulsen

**Affiliations:** 1 Department of Neurosurgery, Odense University Hospital, Odense, Denmark; 2 Clinical Institute, University of Southern Denmark, Odense, Denmark; 3 BRIDGE – Brain Research Inter Disciplinary Guided Excellence, Clinical Institute, University of Southern Denmark, Odense, Denmark; 4 Department of Endocrinology, Odense University Hospital, Odense, Denmark; 5 Department of Pathology, Odense University Hospital, Odense, Denmark

**Keywords:** Neuroscience, Endocrinology, Medical research, Neurology

## Abstract

Endoscopic pituitary surgery has shown promising results. This study reports the experiences of experienced microscopic pituitary surgeons changing to the endoscopic technique, and the beneficial effects on the postoperative outcomes. 45 transsphenoidal endoscopic-assisted surgeries performed in 2016–2017 were compared with 195 microscope-assisted surgeries performed in 2007–2017 for pituitary adenoma. Tumour size, hormonal status and vision were assessed preoperatively and 3–5 months postoperatively. Cases were identified through electronic patient records. GTR was achieved in 39% of the endoscopic operations vs. 22% of microscopic operations, *p* = 0.018. Mean duration of surgery was 86 min (77–95) with the endoscopic technique vs. 106 min (101–111) with the microscopic technique, *p* < 0.001. New hypothalamus–pituitary–adrenal axis deficiencies were observed after 3% of endoscopic vs. 34% microscopic operations, p = 0.001, and overall fewer postoperative pituitary deficiencies were observed in the endoscope-assisted group. Complications within 30 days of surgery occurred in 17% of endoscopic operations vs. 27% of microscopic operations (p > 0.05). Normalization of visual impairment occurred in 37% of the cases with preoperative visual impairment in the endoscopic group vs. 35% of those in the microscopic group (p > 0.05). The endoscopic technique performed better as a surgical procedure for pituitary adenomas. We found no statistically significant differences in complication rate or visual improvement between the two techniques.

## Introduction

Pituitary adenomas (PAs) account for 10–25% of all intracranial tumours^1,2^. They arise from the pituitary in the sella turcica and are classified as either clinically non-functioning pituitary adenomas (NFPAs) or clinically functioning adenomas such as prolactinomas (PRL), adrenocorticotropic hormone (ACTH)-secreting, growth hormone (GH)-secreting or thyroid-stimulating hormone (TSH)-secreting adenomas^3^. PAs can put pressure on adjacent structures, leading to vision field impairment, hypopituitarism and sometimes ophthalmoplegia^4^.

PAs (except PRL) are primarily treated surgically via a transsphenoidal route to the sella turcica^5^. The aims are to remove all tumour tissue (i.e. gross total resection), relieve pressure and minimize the risk of relapse. Both immediate and long-term complications can arise from surgery close to the anterior pituitary cells and stalk, the optic chiasma and adjacent cranial nerves in the cavernous sinus^6,7^. The most common postoperative complications are rhinoliquorrhea (7–15%)^6,8^ and surgically induced hypopituitarism (22%)^7^.

A microscopic approach has been the gold standard for PA surgery for some time. Since Hardy introduced the microscope in 1962^9^, this approach has been refined in attempts to improve complete tumour resection and to minimize postoperative complications^10,11^. Jankowski et al.^12^ demonstrated in 1992 a simpler and faster approach to the sella using an endoscopic transsphenoidal approach that improved the surgeon’s ability to identify high-risk structures and to resect PAs with a supra- and parasellar extension. This endoscopic technique has since been standardized and adopted worldwide as an alternative to the microscopic approach^13,14^. The endoscopic approach has proved promising^15^, also for resection of large adenomas^16^ that invade the supra- and parasellar regions^17^.

While studies have compared the microscopic and endoscopic approaches, the results are inconclusive as to which technique is better. Previous studies have investigated the introduction of the endoscopic procedure into clinical practice and the associated learning curve for surgeons using it as a new technique for transsphenoidal pituitary surgery. These studies all revealed the endoscopic technique to show promising results on gross tumour resection^18,19^, postoperative pituitary function^18,20^, visual field changes^21^ and duration of surgery^22^ except in two studies, where no difference between endoscopic and microscopic pituitary surgery could be detected^23,24^.

The present retrospective study was conducted to further examine the learning curve of neurosurgeons experienced in the microscopic approach who changed to the endoscopic approach. This, combined with detailed studies on the postoperative status, have not previously been thoroughly investigated. We thus compared pre- and postoperative MRI scans, complications within 30 days of surgery, biochemistry, and computer perimetry for patients treated with either the endoscopic or the microscopic technique.

## Results

### Inclusion

Of the 64 transsphenoidal PA procedures performed in 2016 and 2017, four cases had unavailable pre- or postoperative MRI scans. Fifteen operations used the microscopic technique, giving 45 cases in the endoscope-assisted group (Table 1). As previously reported^8^, 180 procedures using the microscopic technique had been performed during 2007–2015. These 180 were added to the 15 cases from 2016–2017 to give 195 in the microscope-assisted group (Table 1).

**Table 1:** Preoperative characteristics of patients with pituitary adenomas undergoing either endoscopic or microscopic surgery.

	Endoscopic	Microscopic
n	45	195
Gender, n (male/female)	25/20	107/88
Median age at surgery (range)	61 years (21–83)	58 years (17–87)
Primary operations/Re-operations, n (%)	36 (80)/9 (20)	173 (89)/21 (11)
**Pituitary function, n (%)**		
HPA-axis		
Intact	30 (67)	121 (64)
Deficient	11 (24)	52 (27)
Cushing’s	3 (7)	11 (6)
Unknown	1 (2)	6 (3)
HPT-axis		
Intact	25 (56)	115 (61)
Deficient	19 (42)	66 (35)
Unknown	1 (2)	4 (2)
HPG-axis		
Intact	20 (44)	81 (43)
Deficient	22 (49)	102 (54)
Unknown	3 (7)	7 (4)
ADH intact (deficient)	42 (3)	166 (26)
**Adenoma subtypes, n (%)**		
Non-functioning pituitary adenoma	29 (66)	115 (63)
GH-secreting	8 (18)	30 (16)
ACTH-secreting	3 (7)	11 (6)
Others	4 (9)	28 (14)
**Vision impairment, n (%)**	90	30
None	36 (40)	119 (31)
Peripheral	38 (42)	225 (58)
Central	4 (4.5)	30 (7.7)

*HPA* Hypothalamus–pituitary–adrenal, *HPT* Hypothalamus–pituitary–thyroid, *HPG* Hypothalamus-pituitary–gonadal, *GH* Growth hormone, *ACTH* Adrenocorticotropic hormone.

### Resection

Gross total resection was achieved in 39% (17/45) of operations using the endoscopic technique compared to 22% (42/195) of operations using the microscopic technique, *p* = 0.018. The mean tumour remnant volume was 2.21 cm^3^ (1.31–3.11) in the endoscopic group vs. 2.44 cm^3^ (1.68–3.21) in the microscope group*, p* = 0.775. The mean resected volume with endoscope vs. microscope was 2.75 cm^3^ (2.08–3.43) vs. 4.90 cm^3^ (4.25–5.54), *p* = 0.002, corresponding to a resection degree of 67% and 68%, respectively (*p* = 0.8876).

**Figure 1:**
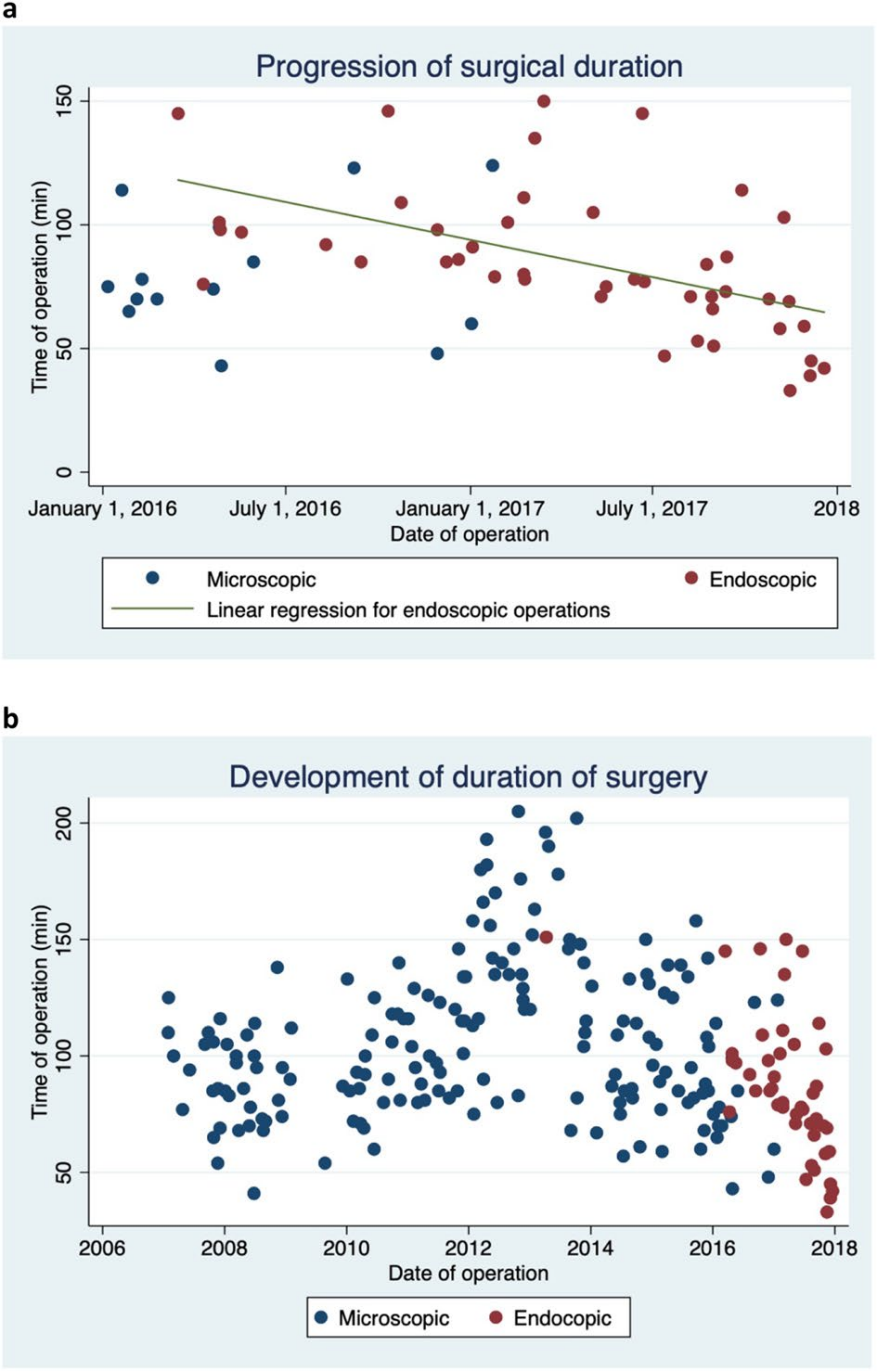
Comparison between endoscopic-assisted and microscopic-assisted surgery for pituitary adenomas. (**a**) The change in duration of surgery from the introduction of the endoscope in 2016, showing a clear learning curve from the start of 2016 to the end of 2017. (**b**) Development in the duration of surgery from 2007 to 2018.

Mean duration of surgery was 86 min (77–95) with the endoscope vs. 106 min (101–111) with the microscope, *p* < 0.001. As shown in Fig. 1, the duration of the endoscopic procedure gradually declined from the time of introduction of the technique to the end of the study period, reflecting the surgeons’ learning curve.

Complications within 30 days of surgery were observed in 17% (8/45) of patients operated with the endoscopic technique compared to 27% (51/195) in the microscope-assisted group *p* = 0.163. When complications were graded according to the classification of Ibańez et al.^18^, grade II complications or higher were seen in 4% (2/45) of the endoscopic group (one with a lung embolus and one with rhinoliquorrhea) compared to 20% (39/195) of the microscopic group (21 with rhinoliquorrhea ), *p* = 0.012 (Table 2).

**Table 2:** Comparison of surgical outcomes and postoperative complications for patients with pituitary adenomas undergoing either endoscopic or microscopic surgery.

	Endoscopic	Microscopic	p-values
**Resection**			
Mean preoperative tumour volume, cm^3^ (95% CI)	4.87 (3.63–6.10)	7.33 (6.31–8.36)	**p = 0.025**
Tumour remnant, n (%)	28 (61)	153 (78)	**p = 0.018**
Mean postoperative tumour volume, cm^3^ (95% CI)	2.21 (1.31–3.11)	2.44 (1.68–3.21)	p = 0.775
Mean resected volume, cm^3^ (95% CI)	2.75 (2.08–3.43)	4.90 (4.25–5.54)	**p = 0.002**
Mean duration of surgery (95% CI)	86 min (77–95)	106 min (101–111)	**p < 0.001**
**Complications, n (%)**			
Complications reported within 30 days	8 (17)	51 (27)	p = 0.163
Verified complications^a^	2 (4)	39 (20)	**p = 0.012**
Lung emboli	1 (2)	4 (2)	p = 0.961
Rhinoliquorrhea	1 (2)	21 (11)	p = 0.067
Meningitis	0	4 (2)	p = 0.326
Haematoma	0	3 (1.5)	p = 0.396
Haemorrhage	0	2 (1)	p = 0.489
Others	0	9 (5)	p = 0.137
**Graded by classification from Ibañez et al.**^18^**, n**			
Grade I	6 (a: 5, b:1)	12 (a: 5, b: 7)	p = 0.099
Grade II	2 (a: 2)	29 (a: 25, b: 4)	p = 0.060
Grade III	0	10 (a: 10)	p = 0.121
Grade IV	0	0	

*p*-values < 0.05 was regarded as stastically significant and highlighted in bold

^a^By CT scan, Lumbar puncture and/or blood tests.

### Pituitary function

**Figure 2:**
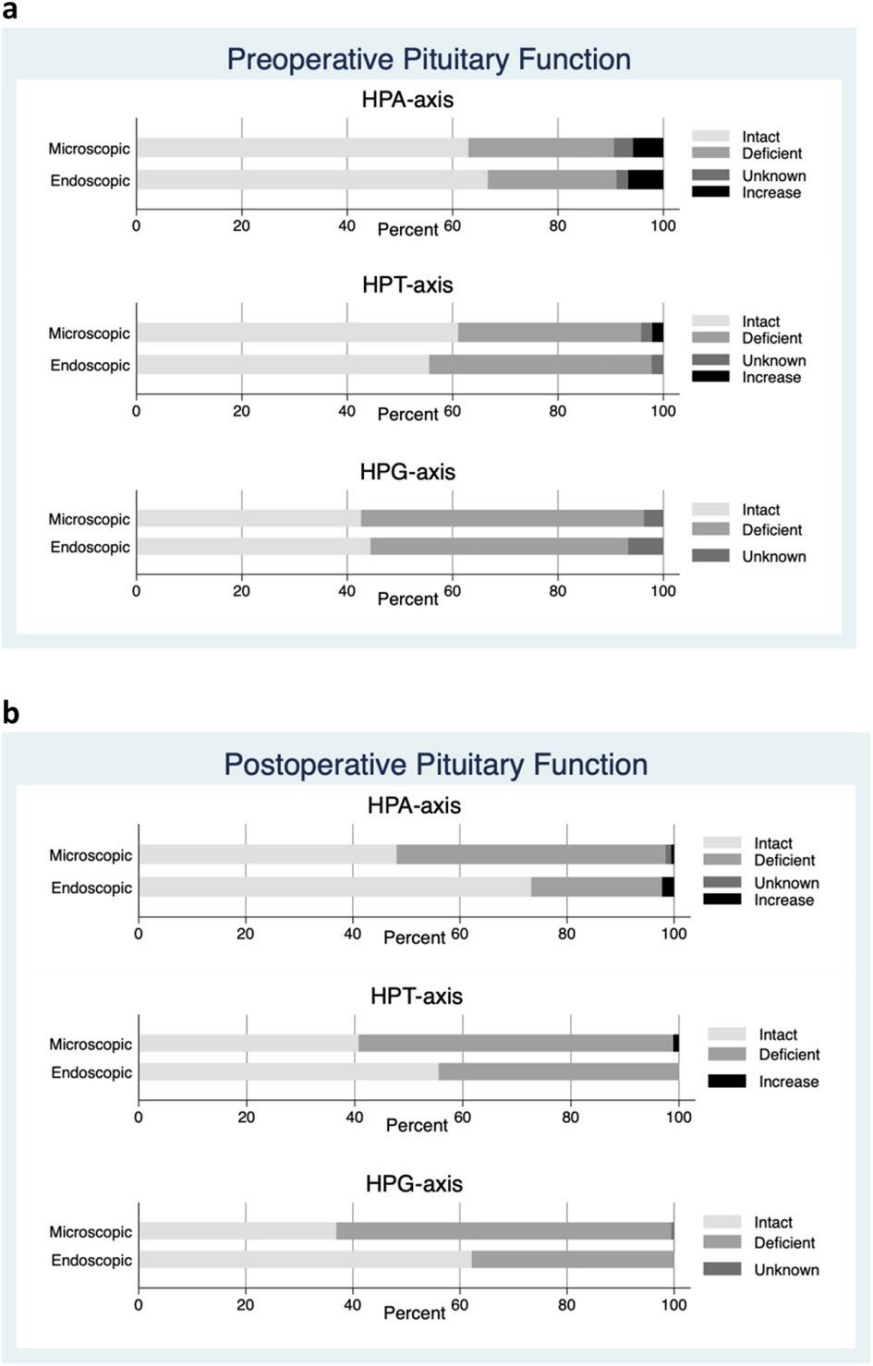
Preoperative (**a**) and postoperative (**b**) clinical biochemical data showing pituitary function in patients with pituitary adenomas undergoing either endoscopic or microscopic surgery.

The HPA-axis was intact preoperatively in 67% of patients in the endoscopic group vs. 64% in the microscopic group (Table 1). New HPA-axis deficiencies developed after surgery in 3% of patients in the endoscopic group and 34% in the microscopic group, *p* = 0.001 (Table 3). HPA-axis function normalized after surgery in 27% of patients in the endoscopic group and 17% in the microscopic group, *p* = 0.444 (Fig. 2).

**Table 3:** Postoperative pituitary function and visual field impairment in patients with pituitary adenomas undergoing endoscopic or microscopic surgery, showing proportion of patient developing new pituitary deficiencies or experiencing normalization of pituitary function (remission).

	New deficiency (%)		p-values	Remission of function (%)		p-values
	Endoscopic	Microscopic		Endoscopic	Microscopic	
**Pituitary function**						
HPA-axis	1/30 (3)	41/120 (34)	**p = 0.001**	3/11 (27)	9/52 (17)	p = 0.444
HPT-axis	4/26 (15)	43/114 (38)	**p = 0.030**	3/15 (16)	3/66 (5)	p = 0.092
HPG-axis	0/20 (0)	30/80 (38)	**p = 0.011**	7/22 (32)	19/102 (19)	p = 0.168
**Visual field impairment**	1/36 (3)	9/119 (7)	p = 0.270	14/38 (37)	79/225 (35)	p = 0.836

*p*-values < 0.05 was regarded as stastically significant and highlighted in bold

The HPT-axis was intact preoperatively in 55% of patients in the endoscopic group vs. 61% in the microscopic group (Table 1). New TSH-dependent deficiencies developed after surgery in 15% of patients in the endoscopic group compared to 38% in the microscopic group, *p* = 0.030 (Table 3). HPT-axis function normalized after surgery in 16% and 5% respectively, *p* = 0.092.

The HPG-axis was intact preoperatively in 44% of patients in the endoscopic group vs. 43% in the microscopic group (Table 1). While no patients in the endoscopic group developed new deficiencies after surgery, 38% of patients in the microscopic group did, p = 0.011 (Table 3). HPG-axis function normalized after surgery in 32% of patients in the endoscopic group compared to 19% in the microscopic group, p = 0.16.

Among the patients with clinically functioning adenomas, 18% (8/45) in the endoscopic group had acromegaly compared to 19% (37/190) in the microscopic group, p = 0.80. Of these, 63% (5/8) in the endoscopic group were biochemically cured from their GH hypersecretion after surgery compared to 49% (18/37) in the microscopic group, p = 0.48. Among the patients with clinical Cushing’s syndrome (Table 1), 66% (2/3) in the endoscopic group showed biochemical remission vs. 91% (10/11) in the microscopic group, p = 0.29.

### Visual field impairment

Visual field assessment by computed perimetry showed that 40% of patients in the endoscopic group had intact vision before surgery compared to 31% in the microscopic group, *p* = 0.03 (Table 1). Surgery-induced visual field impairment was observed in 3% of patients in the endoscope-assisted group and 7% in the microscope-assisted group, *p* = 0.27 (Table 3). Postoperative visual improvement was observed in 37% of patients in the endoscopic group and 35% in the microscopic group, *p* = 0.836 (Table 3).

## Discussion

This retrospective study of transsphenoidal surgery for pituitary adenomas examined the introduction of an endoscope-assisted technique and the results from the first 45 procedures. The findings suggest that introduction of the endoscopic transsphenoidal approach was associated with a relatively steep learning curve for the surgeons, by improving the degree of surgical resection, reducing the duration of the surgical procedure and reducing the overall complication rate compared to the standard microscope-assisted technique. The present study adds to the literature in favour of using the endoscopic technique for transsphenoidal pituitary surgery.

We found that significantly fewer patients undergoing endoscopic surgery had tumour remnants (i.e. a higher rate of gross total resection) compared to those undergoing the microscopic technique. Our findings are similar to previous studies^15,19,20^, but we document for the first time that while the surgeon’s learning curve is steep, the benefits of using the endoscope are apparent early after changing technique.

Duration of surgery was shorter for the endoscopic technique (mean 86 min, 95% CI 77–95) compared to the microscopic technique (mean 106 min, 95% CI 101–111). The learning curve illustrated a steady decrease in duration of surgery from the introduction of the endoscopic technique in 2016. These findings are comparable to previous studies showing shorter duration of surgery with the endoscopic technique^21^.

The mean resected volume was smaller in the endoscopic group (2.75 cm^3^ (2.08–3.43) vs. 4.90 cm^3^ (4.25–5.54), *p* = 0.002). There was, however, no difference in mean postoperative tumor volume. The reason for this lower resected volume in the endoscopic group compared to the microscopic group is probably, that the mean preoperative tumor volume in the microscopic group were larger than in the endoscopic group. We do not have a solid explanation for this except that (1) surgery is recommended on adenomas larger than 20 mm in diameter and (2) there is a general tendency to operate on smaller adenomas after the introduction of the endoscope, especially since the endoscopic procedure at our institution proved more tolerable by the patients.

Only 2% of patients in the endoscopic group were observed with postoperative rhinoliquorrhea compared to 11% in the microscope group. These results are comparable to the findings of Messerer et al.^19^ and Eseonu et al.^21^ and, while not quite statistically significant, they further support the advantage of using the endoscopic technique for transsphenoidal pituitary surgery.

We found that fewer patients operated with the endoscopic technique developed postoperative anterior pituitary gland deficiency compared to the microscopic group. These results are comparable to other studies^19,22^. Postoperative pituitary function is most often omitted in systematic reviews or meta-analyses^15,23^. However, one meta-analysis combined the results from six previous studies to investigate postoperative pituitary function. The results failed to show statistically significant differences between different surgical procedures^16^, only reporting postoperative hypopituitarism in 3% (8/263) in the endoscopic group vs. 6% (16/262) in the microscopic group. This makes us doubt the assessment of pituitary deficiency in these studies. Our data support the tendencies previously reported, i.e. that the endoscopic procedure was gentler and had less effect on pituitary function than the microscopic technique.

The present study did not find a significant difference in the rate of curative surgery for patients with acromegaly or Cushing’s syndrome. Like previous studies, our sample size of secreting adenomas was too low to draw conclusions on this parameter^23^, and further studies focusing on secreting adenomas are needed to clarify this.

We found no difference between the endoscopic and microscopic groups in the proportion of patients with complete recovery of vision after surgery. These findings are comparable to the findings of Muskens et al.^24^. Results vary between publications, however, possibly because postoperative improvement of visual field impairment is greatly influenced by the preoperative duration of optic apparatus compression. As shown by Anik et al.^25^, there are two important phases in visual improvement and its assessment, and the type of examination is important for the results. Furthermore, the timing of evaluation of visual field impairment is important when determining improvements after surgery.

## Conclusion

We introduced the endoscopic transsphenoidal approach to replace the traditional microscopic transsphenoidal approach for pituitary surgery in 2016. The endoscopic approach shortened the duration of surgery and minimized the complication rate including postoperative pituitary dysfunction. In non-functioning pituitary macroadenomas, the endoscopic technique also improved gross total resection rates.

## Methods

### Study design

The inclusion criteria were patients with an NFPA or a clinically functioning adenoma who underwent planned transsphenoidal surgery for PA at our department in 2016 or 2017. The study was retrospective, and patients were identified using the National Danish Patient Registry and ICD-10 diagnostic code D352 (benign pituitary adenoma) combined with surgical codes KAAE10 (transsphenoidal excision of intracranial pathologic tissue) + KZXF95 (intracranial endoscopic technique).

A Karl Storz endoscope was introduced at our department in 2016, and 49 operations were performed in 2016–2017 using the endoscopic transsphenoidal technique. The current study compares the results from these first endoscopic surgeries with the results from a previously published study from our group^8^ reporting on patient outcome after microscope-assisted transsphenoidal pituitary surgery performed from 2007 to 2015. All endoscopic surgical procedures were performed by the same team of neurosurgeons (CBP and FRP) with no assistance from ear-nose-throat surgeons. The microscopic surgeries where performed by FRP and a now retired neurosurgeon.

### Surgical technique

#### Endoscopic technique

After induction of general anaesthesia, cottonoids with Moffett’s solution (a combination of cocaine, sodium bicarbonate and adrenaline given as standard practice in many rhinological procedures to provide local anaesthesia, vasoconstriction and decongestion^26^) were placed for 10–15 min in each nostril. Neuronavigation with magnetic resonance imaging (MRI) and computed tomography (CT) was used to achieve endoscopic access to the sphenoid sinus (0° and 30° endoscopes coupled to a camera, Storz). Using a bi-nostril technique, the adenoma was removed using blunt curettes. Haemostasis was achieved with temporary placement of Spongostan and, if necessary, Surgiflo or FloSeal. The dura was sealed using Adherus (Stryker). Lumbar drainage of cerebrospinal fluid was not used. All patients were admitted to a semi-intensive neurosurgical ward for postoperative monitoring. If there were no signs of early postoperative surgical complications, patients were routinely transferred to the endocrinology unit the following day.

#### Microscopic technique

As described in^8^, after induction of general anaesthesia, a submucosal paraseptal transsphenoidal microsurgical technique was used in all patients using a standard microscope (Zeiss, Pentoro) and neuronavigation (Medtronic) guiding. Adenomas were removed using blunt curettes, and haemostasis was achieved with temporary placement of Spongostan and, if necessary, Surgiflo or FlowSeal. The dura was closed using Tachoseal and, in some cases, the sellar floor was reconstructed using titanium mesh or septal bone. The nasal septum was repositioned and fixed with nasal packing for 12–24 h. Lumbar drainage was not used routinely.

### Rhinoliquorrhea

Treatment of postoperative CSF leakage followed the institution local guidelines: in case of suspicion of early postoperative CSF leakage, the patients were kept in bed with up to 45° elevated head rest for additional three days. If a CSF leakage was still suspected, a lumbar drain was inserted and kept in place with the patient in bed for additional five days. In case the rhinoliquorrhea persisted after removal of the lumbar drain, operative closure was performed using muscle and fat graft.

### Data collection

#### Magnetic resonance imaging and postoperative complications

Knosp (by Micko et al.)^17^ and Hardy (by Hardy and Vezina)^27^ classifications were used to classify adenoma extension based on the patients preoperative MRI (1.5 T or 3 T). Tumour volume was calculated using 3D volumetric analysis in Horos on axial sections of T1-weighted MRI images with contrast. Similarly, tumour remnant volume was calculated on routine follow-up MRI scans performed 4–6 months after surgery. In case of uncertainty about tumour delineation, scans were discussed with senior neurosurgeons. Duration of surgery and complications (rhinoliquorrhea, infection, intracranial haemorrhage and the need for additional surgery) within 30 days were registered. Complications were classified according to Ibañez et al.^18^.

#### Biochemical data

Pituitary hormones were assessed preoperatively and six weeks postoperatively using clinical biochemical data on the hypothalamic–pituitary–somatotropic axis (s-GH and s-IGF-1), the hypothalamic–pituitary–gonadal (HPG) axis (s-FSH, s-LH, s-oestradiol for pre-menopausal women, s-total testosterone for men and s-sex hormone-binding globulin (s-SHBG)), the hypothalamic–pituitary–thyroid (HPT) axis (s-TSH, s-T4 and s-thyroid-binding globulin (s-TBG)) and the hypothalamic–pituitary–adrenal (HPA) axis (s-ACTH and Synacthen test). Deficiency in any axis was defined as biochemical data outside reference values for the specific hormone or if the patient was already on substitution therapy. Intact function was defined as hormone levels within the normal range, whereas increased values of IGF-1, cortisol or T4 led to specific tests for secreting adenomas. During the postoperative hospital stay, patients were monitored for the development of diabetes insipidus (DI) and hypopituitarism.

The exact diagnosis was based on the combination of biochemical data and results from postoperative pathological and immunohistochemical analyses performed by specialized neuropathologists.

#### Visual field impairment

Vision was evaluated pre- and postoperatively in all patients able to cooperate. Visual field was evaluated by quadrant affected (upper-lower temporal/upper-lower nasal) combined with mean deviation in decibel for each eye. Central visual impairment was determined by rapidly declined vision on the Snellen chart or blindness. Based on the mean deviations, vision in each eye was categorized as intact vision, peripheral field impairment or central visual impairment.

### Statistical analysis

Data were entered into a REDCap database (a secure web application for building and managing online surveys and databases) with assistance from OPEN (Open Patient data Explorative Network) and were analysed using Student’s *t*-test and chi-squared test (STATA/IC 15.0). *p*-values < 0.05 was regarded as stastically significant.

### Ethics

The study was approved by the Danish Patient Safety Authority (ID 3-3013-2400/1) and the Danish Data Protection Agency (ID 18/51314). Since there were no direct patient contact related to this retrospective study, Danish legislation does not require permission from individual patients.

This study on humans were carried out in accordance with guidelines and regulations from the Danish Health Act § 46.2.
